# Prognostic value of secreted phosphoprotein-1 in pleural effusion associated with non-small cell lung cancer

**DOI:** 10.1186/1471-2407-14-280

**Published:** 2014-04-23

**Authors:** He Zhang, Hong-bing Liu, Dong-mei Yuan, Zhao-feng Wang, Yun-fen Wang, Yong Song

**Affiliations:** 1 Department of Respiratory Medicine, Jinling Hospital, Nanjing University School of Medicine, 305 East Zhongshan Road, 210002 Nanjing, Jiangsu Province, China; 2 Department of Respiratory Medicine, Yijishan Hospital, Wannan Medical College, Wuhu, China

**Keywords:** Secreted phosphoprotein-1, Osteopontin, Wmalignant pleural effusion, Non-small cell lung cancer, Prognosis

## Abstract

**Background:**

Malignant pleural effusion (MPE) is a common complication of advanced lung cancer. Research has shown that secreted phosphoprotein-1 (SPP1) is essential in MPE associated with lung cancer. This retrospective study was performed to evaluate the prognostic significance of SPP1 in the MPE of patients with non-small cell lung cancer (NSCLC).

**Methods:**

MPE specimens were obtained from 85 NSCLC patients (study group), and pleural effusion specimens were obtained from 24 patients with benign lung disease (control group). Specimens were tested for SPP1 using enzyme-linked immunosorbent assay (ELISA). Based on the cutoff value of receiver operating characteristic (ROC) curve analysis, the study patients were divided into a high-SPP1-expression subgroup and a low-expression subgroup. The primary and secondary endpoints of this study were progression-free survival (PFS) and overall survival (OS).

**Results:**

The SPP1 levels of the study group were significantly higher compared to those of the controls (Mann–Whitney *U* test, *P* = 0.017). The number of extrapulmonary metastases was significantly higher in the high-SPP1-expressing patients than in the low-expressing patients (*P* = 0.03). Kaplan-Meier survival analysis showed that SPP1 levels were negatively associated with OS and PFS in both subgroups of study patients (*P* = 0.026; *P* = 0.039, respectively). Cox regression analysis showed that SPP1 was an independent prognostic factor in patients with NSCLC (HR = 1.832, 95% confidence interval: 1.003–3.345; *P* = 0.049).

**Conclusion:**

SPP1 in pleural effusion can be used for the auxiliary diagnosis of MPE and used to determine the prognosis of patients with NSCLC.

## Background

The incidence and mortality of lung cancer remain obstinately high with the environment deterioration and the population aging [1,2]. Malignant pleural effusion (MPE), a common complication of advanced lung cancer, severely impedes respiration and circulation, affecting patients’ life quality [3] with greatly shortened survival period [4]. Currently, diagnosis accuracy and treatment efficacy for MPE are still to be improved [5], and individual tumor markers such as carcinoembryonic antigen (CEA) and cytokeratin 19 fragments (CYFRA 21–1) in making diagnosis is also unsatisfactory [6,7]. Therefore, further study on MPE is necessary for reliable diagnostic methods.

Secreted phosphoprotein 1 (SPP1), also known as osteopontin, is a secreted arginine-glycine-aspartic acid (RGD)-containing phosphorylated glycoprotein with a molecular weight of about 325,000 [8]. It was first isolated as an extracellular matrix (ECM) protein from bone matrix [9]. The human *SPP1* gene locates on chromosome 4 (4q13) with 7 exons and 6 introns [10]. The protein can be secreted by various cells, including osteoclasts, macrophages, epithelial cells, endothelial cells [11,12]. In addition, tumor cells can also secrete soluble SPP1, which is different from the matrix protein [13].

The expression of SPP1 is strongly associated with tumorigenesis and tumor metastasis. It has been noted that the expression of SPP1 is high in numerous tumors, including lung cancer [14,15]. Ongoing research has discovered that SPP1 in MPE due to lung cancer has numerous effects: it progresses the tumor both in its growing and metastasis, and prolongs the survival of cancer cells [16]. Moreover, SPP1 increases vascular permeability. Cui et al. found that SPP1 could increase pleural vascular permeability by inducing vascular endothelial growth factor (VEGF) expression [17]. Additional studies have confirmed that SPP1 has a non-VEGF-dependent effect on vascular permeability as well. Also, SPP1 promotes intrapleural cancer dissemination [18]. The invasion of ECM by tumor cells may lead to conditions favorable for metastasis [19]. In an in vitro study, Hu et al. found that SPP1 promotes invasion of ECM by NSCLC cells, and its effect may be blocked by SPP1 antibodies [20]. Therefore, SPP1 is essential in the formation and development of MPE.

To the best of our knowledge, however, there have not been any studies on the relationship between SPP1 in MPE and the diagnosis and prognosis of lung cancer. So this study is performed to investigate whether the expression of SPP1 in MPE associated with NSCLC are useful for NSCLC in making diagnosis and prognosis.

## Methods

### Study participants

85 NSCLC patients, 43 men and 42 women, aged 36–86 years, with a mean age of 64, treated at Jinling Hospital (Nanjing, Jiangsu Province, China) from January 1, 2010 to December 31, 2012, were retrospectively enrolled for the trials. NSCLC was confirmed by either exfoliative cytology of pleural fluid or pleural biopsy. All pleural fluids were confirmed MPE by exfoliative cytology of pleural fluid, 7 and some patients received pleural biopsy for their histopathology. The malignancies included 13 squamous cell carcinomas, 67 adenocarcinomas, 1 adenosquamous carcinoma, and 4 unknown cancers (malignant cells were noted in the exfoliative cytology of pleural fluid, and the pathological features resembled NSCLC but the subtypes could not be confirmed) (Table 1). All the lung cancer patients received at least 2 cycles of platinum-based, standard first-line chemotherapy or targeted therapy. All study patients had stage IV cancer according to the 7th edition of the AJCC/UICC TNM system. The treatment efficacy was accessed following the Response Evaluation Criteria in Solid Tumors (RECIST) 1.1.

**Table 1:** The characteristic between study group and case–control group

**Characteristic**	**Study group**	**Control group**
Number	85	24
Gender		
Male	43	14
Female	42	10
Mean Age (range)	64 (36–86)	61 (23–86)
Disease	Non-small cell lung cancer	Benign lung disease
	Adenocarcinoma 67	Pulmonary tuberculosis 18
	Squamous cell carcinoma 13	Lung infection 2
	Adenosquamous carcinoma 1	Pulmonary aspergillosis 2
	Unkown-subtype* 4	AECOPD^ 2

*Unknown-subtype: malignant cells were found in the exfoliative cytology of pleural fluid, and the pathological features resembled non-small cell lung cancer but could not be definitively identified its subtype. ^AECOPD: acute exacerbation of chronic obstructive pulmonary disease.

24 pathologically confirmed benign lung disease patients, 14 men and 10 women, aged 23–86 years, with a mean age of 61, were taken as controls. There were 18 patients with tuberculous pleurisy, 2 with acute exacerbation of chronic obstructive pulmonary disease, 2 with lung infections, and 2 with pulmonary aspergillosis (Table 1).

### Pleural fluid specimens

Both malignant and nonmalignant pleural effusion specimens were collected from the study patients and controls respectively. Follow-up of the study patients began from the date of specimen collection. The follow-up ended until patient death or the follow-up period finish on January 31, 2013. Five patients were lost to follow-up. The progression-free survival for MPE was defined as from the time diagnosed with pleural effusion to significant progression. Significant progression are confirmed in those with target lesions in solid tumors considered as progression according to RECIST 1.1 and the malignant pleural fluid volume is rising rapidly compare with its baseline during therapy. All patients signed informed consent and the study was approved by Ethics Committee of Jinling Hospital.

Fresh pleural fluid specimens were collected from patients and kept in 10 ml centrifugal tubes without additive solution and centrifuged at 1000 × g for 15 minutes within 30 minutes after collection, and then stored in Eppendorf tubes in a -20°C freezer until analysis.

The specimens were placed at room temperature for 5 minutes for thawing. There is no dilution criterion about samples of pleural fluid according to the directions of ELISA kit (DOST00, R&D System Company, Minneapolis, MN, USA). We randomly take 1 piece of sample from both study group and case–control group, then took the OPN Standard (200 ng/ml) from the ELISA kit using the Calibrator Diluent RD5-24 (DOST00, R&D System Company, Minneapolis, MN, USA) to produce 1:5, 1:10, 1:50 and 1:100 diluted samples to run a mini pilot experiment. With the Calibrator Diluent RD5-24 served as the zero standard (0 ng/ml), the result showed that samples from either study group or case–control group which diluted in 100-fold were well consistent with the OPN Standard performed in OD values. Based on the results of this pilot experiment, all the specimens were diluted 100-fold with Calibrator Diluent RD5-24 before low-speed centrifugation (100 × g) for 3 min. The specimens were assayed using the Human Osteopontin (OPN) Immunoassay Quantikine ELISA kit (DOST00, R&D System Company, Minneapolis, MN, USA) following the manufacturer’s instructions. The results were read on a microplate reader (ELx808, Bio-Tek Instruments,Winooski, VT, USA) at 450 nm, with a correction wavelength at 540 nm). The corresponding OD values were obtained.

### Statistical analysis

A point-to-point calculation was performed to establish the standard curve (r = 0.99992). The SPP1 concentrations were calculated from the absorbances. The SPP1 concentrations of the study patients were compared to those of the control patients using the Mann–Whitney *U* test and the results were shown as median and quartile interval. A cut-off value, selected by receiver operating characteristic curve (ROC) analysis, was used to divide the patients from the study group into 2 subgroups: low-SPP1-expressing patients with SPP1 concentrations lower than the cut-off value; and high-SPP1-expressing patients with SPP1 concentrations not lower than the cut-off value. The differences in gender, age, smoking history, tumor pathology, regional lymph node stage, and number of extrapulmonary metastases of the SPP1 high- and low-expressing patients were analyzed using chi-square test. The PFS and OS of the patients were compared using Kaplan-Meier survival analysis. Prognostic factors were analyzed using Cox regression analysis. A *P-*value of <0.05 was considered statistically significant.

## Results

### SPP1 concentrations

**Figure 1:**
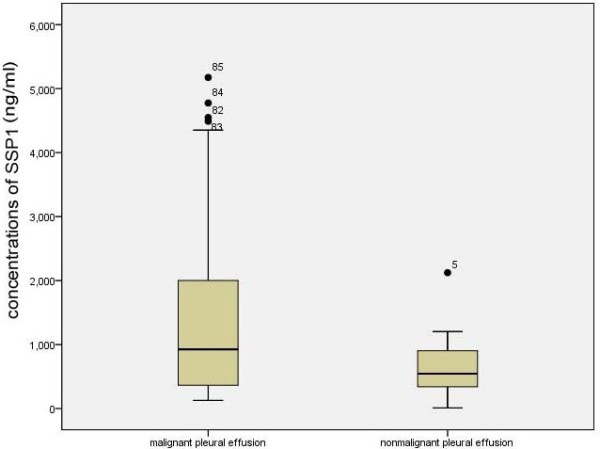
The box plot of concentrations of SSP1 of malignant pleural effusion and nonmalignant pleural effusion.

SPP1 expression was detected in all 109 pleural effusion specimens (Figure 1). The SPP1 concentration in study group and case–control group were tested by Kolmogorov-Smirnov test separately to examine their distribution. The SPP1 levels in case–control group were normally distributed (*P* = 0.860) while in study group were not normally distributed (*P* = 0.004). The SPP1 concentration was significantly higher in the study patients (median = 925.55 ng/ml and quartile interval = 1651.06 ng/ml) than in the control patients (median = 544.89 ng/ml and quartile interval = 574.11 ng/ml) (Mann–Whitney *U* test P = 0.017). The median concentration of SPP1 was about 1.7-fold higher in the study patients than in the control patients.

### Value of SPP1 for the diagnosis of MPE

**Figure 2:**
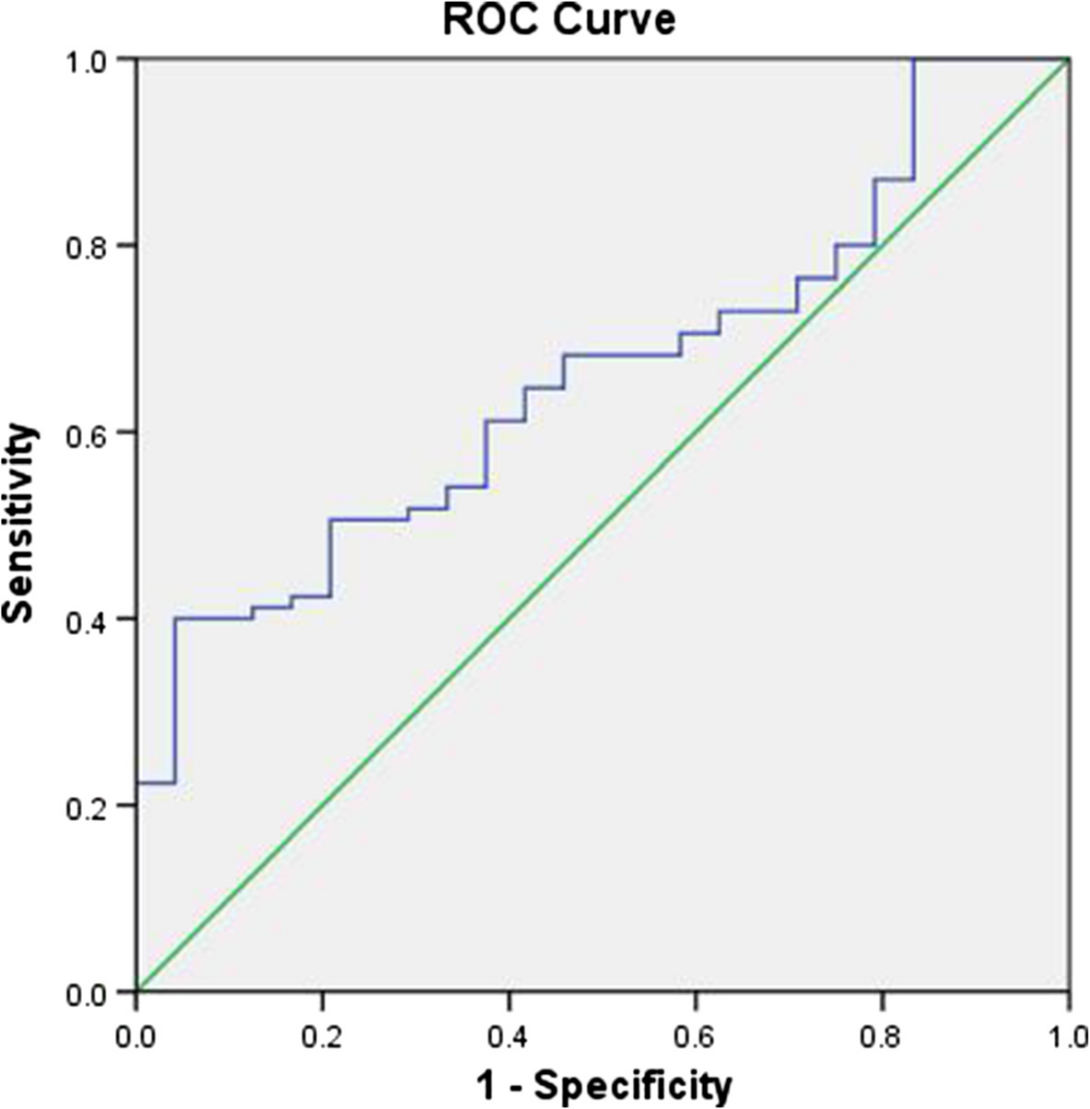
**ROC curve of the SPP1 values in pleural effusion.** SPP1 in PF from all 109 patients are 24 suffered from benign lung disease and 85 with NSCLC. The cut-off value was calculated to be 1247.90 ng/ml (sensitivity, 40.3%; specificity, 95.8%; area under the curve (AUC), 0.660; 95% confidence interval (CI): 0.550-0 .770). Based on cut-off value the positive predictive value is 97.1%; negative predictive value is 31.1%.

The ROC curve was made according to the SPP1 levels of both patients groups (Figure 2). The cut-off value was 1247.90 ng/ml (sensitivity 40.3%; specificity 95.8%; area under the curve (AUC) 0.660; 95% confidence interval (CI): 0.550-0.770), indicating that the SPP1 concentration in pleural effusion may have a role in the auxiliary diagnosis of MPE. High expression of SPP1 implicates high diagnostic specificity. Patient with high SPP1 is strongly suggestive of MPE.

### SPP1 levels in MPE and clinical features

High-SPP1-expression was noted in 34 patients, 14 men and 20 women, 2 with squamous cell carcinoma, 28 with adenocarcinoma, 1 with adenosquamous carcinoma, and 3 with unknown cancers. Low-SPP1-expression was noted in 51 patients, 29 men and 22 women, 11 with squamous cell carcinoma, 39 with adenocarcinoma and 1 with unknown cancer. The Pearson chi-square test showed that gender, age (≤60 years old vs > 60 years old), smoking history (<20 pack year vs ≥ 20 pack year), histological type of lung cancer (adenocarcinoma vs squamous cell carcinoma), and regional lymph node stage (≤N1 vs > N1) were not significantly different between the low- and high-expressing patients (*P* = 0.156, *P* = 0.854, *P* = 0.318, *P* = 0.072, and *P* = 0.164, respectively). The high-SPP1-expressing patients had a significantly greater number of extrapulmonary metastases than the low-expressing patients (*P* = 0.03), suggesting that SPP1 levels in the pleural effusion of NSCLC patients were positively correlated with the number of extrapulmonary metastases (Table 2).

**Table 2:** The relationship between SPP1 expression level and different clinical and pathological features

**Characteristics**	**Total**		Low-SPP1*		**High-SPP1**		***P***
	**NO.**	**%**	**NO.**	**%**	**NO.**	**%**	
Gender							0.156
Men	43	51	29	56	14	41.2	
Women	42	49	22	44	20	58.8	
Age							0.854
≤60	31	36.5	19	37.3	12	35.3	
>60	54	63.5	32	62.7	22	64.7	
Smoking History							0.318
<20	52	61.2	29	56.9	23	67.6	
≥20	33	38.8	22	43.1	11	32.4	
Histology							0.072
Adenocarcinoma	67	78.8	39	76.5	28	82.4	
Squamous	13	15.3	11	21.6	2	5.9	
Adenosquamous	1	1.2	0	0	1	2.9	
Unknown	4	4.7	1	1.9	3	8.8	
Lymphatic Metastasis							0.164
≤N1	14	16.5	6	11.8	8	23.5	
>N1	70	82.4	44	86.3	26	76.5	
Unknown	1	1.1	1	1.9	0	0	
Metastasis organ							0.03
0	27	34.4	21	42.4	6	21.6	
≥1	53	57.3	28	50.8	25	67.6	
Unknown	5	8.3	2	6.8	3	10.8	

*SPP1: secreted phosphoprotein-1.

### SPP1 in MPE and the prognosis of patients with NSCLC

**Figure 3:**
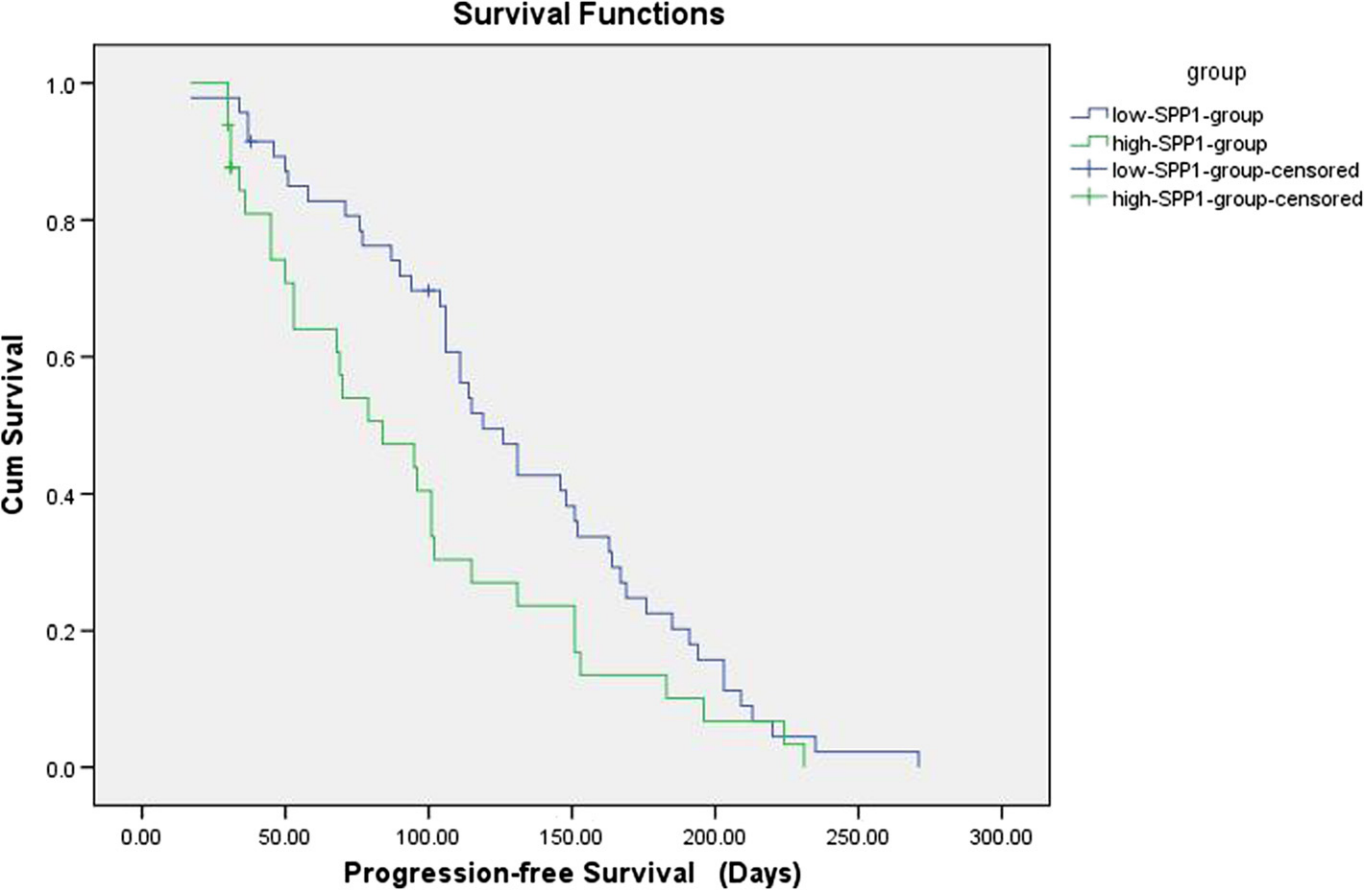
**Kaplan-Meier survival analysis of 85 patients with MPE due to NSCLC.** The median progression-free survival (PFS) was significantly longer for the low-SPP1-expressing patients (119 days) than for the high-expressing patients (84 days) (*P* = 0.039). CUM survival: cumulative survival; Survival Functions: the function of Kaplan-Meier survival analysis.

**Figure 4:**
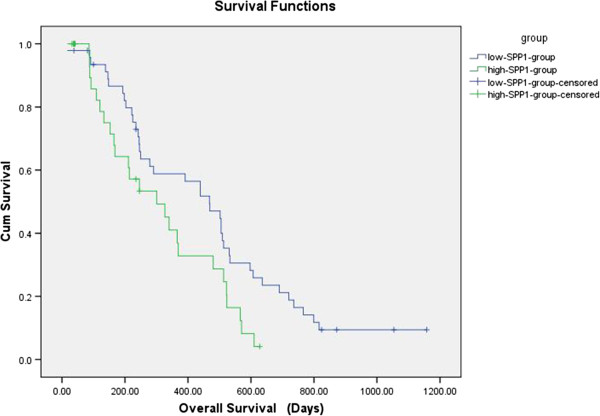
**Kaplan-Meier survival analysis of 85 patients with MPE due to NSCLC.** The median overall survival (OS) was also significantly longer for the low-expressing patients (468 days) than for the high-expressing patients (301 days) (*P* = 0.026). CUM survival: cumulative survival; Survival Functions: the function of Kaplan-Meier survival analysis.

The low-SPP1-expressing patients has significantly longer median PFS (119 days) than that of high-expressing patients (84 days) (*P* = 0.039) (Figure 3), and so it is with the median OS (468 days) than for the high-expressing patients (301 days) (*P* = 0.026) (Figure 4). The results suggest that SPP1 levels in the MPE of patients with NSCLC were negatively correlated with prognosis.

Multivariate survival analysis using the Cox regression model, which evaluated 7 factors, including gender, age (≤60 years vs > 60 years), smoking history (<20 pack year vs ≥ 20 pack year), histological type of lung cancer (adenocarcinoma vs squamous cell carcinoma vs adenosquamous carcinoma), regional lymph node stage (≤N1 vs > N1), number of extrapulmonary metastases, and SPP1 in MPE (low-SPP1-expressing vs high-SPP1-expressing), showed that SPP1 was an independent prognostic factor in patients with MPE associated with NSCLC (hazard ratio = 1.832, 95% CI = 1.003-3.345; *P* = 0.049) (Table 3).

**Table 3:** Multivariate Cox regression analysis of prognostic factors in NSCLC

**Variable**	***P***	HR*	**95.0% CI for HR**	
			**Lower**	**Upper**
Gender	0.775	1.098	0.579	2.082
Age	0.951	1.019	0.555	1.873
Smoking History	0.273	0.681	0.392	1.159
Histology	0.387	1.499	0.599	3.749
Lymphatic Metastasis	0.388	1.405	0.649	3.042
Metastasis Organ	0.734	0.894	0.469	1.706
SPP1^ Groups	0.049	1.832	1.003	3.345

*HR: hazard ratio; ^SPP1: secreted phosphoprotein-1.

## Discussion

In this study, the concentration of SPP1 in pleural effusion samples from 109 patients was determined by ELISA. SPP1 concentrations were significantly higher in the MPE patients than those in the control patients. Based on the cutoff value, the higher the SPP1 levels in pleural effusion specimens were, the lower the false diagnosis rate of MPE would be. In addition, SPP1 levels in MPE specimens were positively correlated with the number of extrapulmonary metastases, and the prognosis of patients was significantly better in the low-SPP1-expressing patients than in the high-expressing patients. SPP1 in MPE was an independent prognostic factor of NSCLC-induced MPE, indicating that SPP1 in pleural effusion can be used for the auxiliary diagnosis of NSCLC-induced MPE, and it may be a prognostic indicator for patient survival and risk of extrapulmonary metastases.

Few investigators have studied the diagnostic and prognostic value of SPP1 in MPE associated with lung cancer, but data on SPP1 on cancer progression is increasing in recent years. Neal et al. found that patients with colon cancer, breast cancer, prostate cancer, and lung cancer had increased SPP1 levels in serum compared with healthy controls; it was almost 2 times higher in patients with lung cancers. Schneider et al. determined the expression levels of SPP1 and osteonectin (ON) in lung cancer tissues using quantitative reverse-transcription polymerase chain reaction and found that it was the increased expression of SPP1 rather than ON was associated with poor prognosis [21]. By testing SPP1 levels in stage I-IIIA NSCLC tissues using immunohistochemistry, and follow-ups for patient outcomes, Italian investigators found that Stage I patients with high expression of SPP1 had poorer prognosis than those with low expression of SPP1 [22]. Mack et al. assessed plasma SPP1 levels of 56 patients with advanced NSCLC who received paclitaxel + carboplatin chemotherapy in the clinical SWOG Study S0003 and continuously monitored plasma SPP1 levels and the treatment response of patients during cycles of chemotherapy. They found that plasma SPP1 levels were significantly associated with treatment response, PFS and OS. Patients with low plasma SPP1 levels had better OS and PFS than those with high levels [23]. Our results are in consistent with these previous studies; however, those studies did not analyze the association between SPP1 level and different clinical features, they and investigated samples of tumor tissue or blood instead of pleural effusion.

Some of the findings on SPP1 concentrations in MPE have been studied. Morsi et al. determined the SPP1 concentrations of serum and pleural effusion from 80 patients (20 with transudative effusion; 30 with MPE, including 14 patients with NSCLC; 30 with tuberculous effusion) using ELISA. Their findings suggested that there was minimal difference between the serum SPP1 levels and effusion/serum SPP1 ratios of the patients with MPE and those with tuberculous effusion [24]. However, Moschos et al. measured serum and pleural effusion levels of SPP1 from 109 patients (42 with MPE, including 19 patients with NSCLC; and 67 with benign diseases) using ELISA, and found that patients with MPE had higher pleural effusion levels and lower serum levels of SPP1 than those with benign disease [25]. Our study showed that SPP1 levels in MPE were significantly higher in the study group than those of the controls. We hypothesize that the findings of Morsi et al. might be attributed to the heterogeneity in malignant effusion as well as less patients were with MPE. The tumor burden and metastases of the sort of group was smaller than the remaining cases. The rest 12 cases are not lung cancer patients, which could lead to larger secretory differentiation and less patients with MPE.

Although novel technologies are continually being introduced, pleural fluid analysis is still inadequate for the diagnosis of MPE. The diagnostic characteristics of single tumor marker in pleural effusion has been reported as follows: CEA (sensitivity 45.9%; specificity 97.0%), CA125 (sensitivity 48%; specificity 85%), CA15-3 (sensitivity 51%; specificity 96%), CA19-9 (sensitivity 25%; specificity 96%) and CYFRA21-1 (sensitivity 55%; specificity 91%) [6,7]. In this study, we have shown that SPP1 in pleural effusion has diagnostic characteristics for MPE similar to those of CEA in pleural fluid.

In addition, our results suggested that the level of SPP1 in MPE was an independent prognostic factor for NSCLC. Both PFS and OS were significantly better in the low-SPP1-expressing patients than in the high-expressing patients. Chi-square analysis showed that SPP1 levels were positively correlated with the number of extrapulmonary metastases, indicating that patients with high levels of SPP1 expression had higher risk of extrapulmonary metastasis. This finding is consistent with the results from a previous study showing that SPP1 can promote tumor growth and metastasis [11]. As previously mentioned, high SPP1 concentrations can inhibit apoptosis of pleural tumor cells and promote intrapleural tumor dissemination. We also conjecture that high levels of SPP1 may prompts tumor cells invasion of the ECM into parietal pleura, proceeding to intravasation of tumor cells into the blood stream and subsequent extravasation assisted with urokinase plasminogen activator (uPA) and matrix metalloproteases (MMPs), leading to migration of neoplastic cells to form metastasis at secondary sites. In conclusion, high concentrations of SPP1 in pleural fluid (especially ≥ 1247.9 ng/ml) are more dangerous and aggressive.

In this study, we evaluated the value of SPP1 as an indicator for the auxiliary diagnosis of MPE and for the prognosis of patients with NSCLC. The cutoff value determined in this study is specific but not sensitive enough, which might have resulted in false-negative results in patients with low SPP1 levels. The reasons for low sensitivity may be the following: a) Interindividual differences may be enhanced when the sample amount is small; b) SPP1 is also related to the formation of lung granulomas because of its role in cellular immunity [26], as research has shown that circulating SPP1 levels in patients with pulmonary tuberculosis were significantly higher than in healthy controls, while the levels might reflect the activity of tuberculosis [27]; and c) SPP1 levels in exudative pleural effusion (e.g., tuberculous pleural effusion) were significantly higher than those in transudative hydrothorax [24,25]. There is a high prevalence of tuberculous pleurisy in China, and patients with tuberculous pleurisy in our study accounted for as many as 75% of together with the control patients, while patients with transudative hydrothorax only accounted for 8.3%. The control group was composed of patients whose disease were not well-controlled, either inflammation originated from pulmonary or granulomatous diseases could be disseminated to pleural space in non-malignant patients and then followed the pleural fluid from the histopathological lesion. This may have accounted for a high level of SPP1 in the control patients, and the value for the controls overlapped the value for the study patients with low expression levels of SPP1. This would lead to reduced sensitivity of the cutoff value.

Our study has inevitable limitations because of various cancers that induce MPE are dramatically different in cellular morphology and biological behaviors. Therefore, further studies with larger sample sizes and optimized pathological types of cancer are needed to verify our findings. We could not measure the corresponding serum samples because the serum samples were not adequate to match all of pleural fluid in the study. This needs valid research in future studies.

## Conclusion

In summary, our study suggests that quantitative determination of SPP1 concentrations in pleural effusions may provide an auxiliary diagnostic modality for differentiating between benign and malignant pleural effusion. An increased level (≥1247.90 ng/ml) in MPE may be an indicator of poor prognosis and risky for distant metastasis. This approach is simple and feasible. Further study is needed because the pathogenesis of MPE is complicated and the pathological mechanisms of association between SPP1 and MPE remain unclear.

## Abbreviations

MPE: Malignant pleural effusion; SPP1: Secreted phosphoprotein-1; ROC: Receiver operating characteristic; PFS: Progression-free survival; OS: Overall survival; CEA: Carcinoembryonic antigen; VEGF: Vascular endothelial growth factor.

## Competing interests

All authors disclosed no potential conflicts of interests.

## Authors’ contributions

HZ, HL, DY, ZW, YW, YS have made substantial contributions to conception and design of the study. HZ, HL carried out acquisition of data. HZ, DY, ZW, YWcarried out analysis and interpretation of data. HZ, HL have been involved in drafting the manuscript. All authors read and approved the final manuscript.

## Pre-publication history

The pre-publication history for this paper can be accessed here:

http://www.biomedcentral.com/1471-2407/14/280/prepub
